# Bioactive Enrichment and Sustainable Processing of Vegetable Oils: New Frontiers in Agri-Food Technology

**DOI:** 10.3390/foods14050769

**Published:** 2025-02-24

**Authors:** Sandra Montoro-Alonso, Xavier Expósito-Almellón, Daniel Martínez-Baena, Joana Martínez-Martí, Ascensión Rueda-Robles, Raúl Pérez-Gálvez, Rosa Quirantes-Piné, Jesús Lozano-Sánchez

**Affiliations:** 1 Department of Food Science and Nutrition, University of Granada, Campus Universitario s/n, 18071 Granada, Spain; sandramontoro@correo.ugr.es (S.M.-A.); xaviexposito14@correo.ugr.es (X.E.-A.); danimb@ugr.es (D.M.-B.); joamarm8@ugr.es (J.M.-M.); jesusls@ugr.es (J.L.-S.); 2 Food Chemistry and Microstructure Research Group, Instituto Universitario de Ingeniería de Alimentos–FoodUPV, Universitat Politècnica de València, Camí de Vera s/n, 46022 València, Spain; 3 Department of Chemical Engineering, Faculty of Sciences, University of Granada, Campus Fuentenueva s/n, 18071 Granada, Spain; 4 Department of Analytical Chemistry, Faculty of Sciences, University of Granada, Campus Fuentenueva s/n, 18071 Granada, Spain; rquirantes@ugr.es

**Keywords:** vegetable oils, bioactive compounds, phenolics, advanced extraction technologies, emulsion

## Abstract

Vegetable oils are highly valued for their nutritional and functional properties, driving scientific interest in developing innovative technologies to enhance production processes. These advancements aim to improve yield, nutritional profiles and organoleptic and functional characteristics. Additionally, vegetable oils have been recognised for their ability to incorporate phenolics as bioactive compounds through stabilisation methods, further enhancing their health benefits. This study conducts a systematic review addressing two main objectives: (i) advanced technologies intended to enhance extraction efficiency while improving the overall quality of vegetable oils and (ii) stabilisation strategies developed to enrich and fortify edible vegetable oils with special focus on phenolic compounds. The Preferred Reporting Items for Systematic Reviews and Meta-Analyses (PRISMA) methodology was applied to evaluate their applications in developing bioactive vegetable oil ingredients and foods. Extraction techniques were assessed based on efficiency in yield and their impact on nutritional, organoleptic and functional properties. Pulsed electric field technology emerged as the most promising approach, offering an optimal balance between oil yield and quality. Combining stirring or high-performance dispersion with ultrasound proved effective in forming stable emulsions for phenolic stabilisation. These strategies provide valuable insights for the agro-industrial sector to enhance production processes and develop healthier, bioactive vegetable oils.

## 1. Introduction

The growth in research focused on the safety and sustainability of food processing is a consequence of two factors: advancements in the understanding of food components and their health-promoting properties and improved consumer access to information. These developments demonstrate an increasing consumer preference for products derived from natural ingredients that are perceived to provide high nutritional value and significant health benefits. In response to these trends, the food industry has adopted innovation as a fundamental aspect of strategic development. New production technologies are being developed and implemented to ensure safer and more sustainable practices and strategies to promote healthier dietary habits are gaining momentum. These combined efforts aim to address consumer demands for nutritious and health-enhancing food products, paving the way for a more sustainable and health-conscious future. In this context, phenolic compounds—secondary metabolites naturally present in a wide variety of plants and renowned for their potent antioxidant and anti-inflammatory properties—stand out as a promising tool. Their well-documented roles in promoting health and preventing disease [1,2], coupled with their potential to be incorporated into the formulation of functional foods [3,4], position them as key contributors to the development of healthier and more nutritionally beneficial dietary options. Functional foods, in turn, provide an effective means to enhance the intake of these biologically active compounds, which exert specific health-promoting effects that extend beyond essential nutritional value, addressing both consumer expectations and public health goals [5].

In this context, the vegetable oil industry has developed innovative technologies to produce functional food products. Oil extraction from plant sources (e.g., olive, sunflower, rapeseed) has traditionally been carried out through mechanical pressing and organic solvents. These processes require a significant input of energy, are inefficient in retaining the nutritional properties of the material and generate by-products that present environmental challenges [6]. Moreover, conventional extraction methods may impair the quality of the extracted oil. In the case of olive oil extraction, disruption of the cellular structures of the fruit during milling results in the release of oil from the vacuoles of the mesocarp cells. Nevertheless, the aforementioned procedure also gives rise to the liberation of enzymes and other substrates that have been in contact with the oil [7].

The extraction technique significantly influences the final quality of the oil, with temperature being a particularly important factor. Two traditional methods for extracting seed oil are cold-pressing and hot-pressing. The process of cold-pressing employs relatively mild temperatures, which prevents the formation of harmful components such as trans fatty acids or polar compounds. However, the yield of the extracted oil is lower, which also results in a less intense flavour. In contrast, the hot-pressing method has proved to enhance both the extraction yield and the palatability of the oil, although it typically results in a darker oil colour. Furthermore, the potential for further utilisation of the oil press cake is limited in the case of hot-pressing due to the degradation of albuminous compounds at elevated temperatures [8].

Over the past few decades, a great deal of research has been conducted into new alternatives and advanced methods for oil extraction, which has enabled the formulation of new functional products derived from edible vegetable oils [9]. In this sense, pulsed electric field extraction (PEF), ultrasound-assisted extraction (UAE), high-hydrostatic pressure (HHP), enzyme-assisted aqueous extraction (EAE) and subcritical water extraction (SWE) have emerged as promising techniques for this purpose [10,11,12,13]. The application of novel technologies offers significant advantages over traditional processing in terms of energy usage, time, throughput and the nutritional quality of edible oils [14].

Conversely, the growing consumer interest in healthy nutrition has prompted the development of new food products containing functional ingredients, such as phenolic compounds. This trend has led to the development of new food processing technologies that facilitate the extraction of these functional compounds and their incorporation into diverse food matrices, such as edible oils. This is particularly challenging due to the variable properties of functional ingredients and their interaction with the oil components. The development of specific delivery systems, such as emulsions (micro and nano), has been a key area of research in this field. These systems have been successfully employed to fortify edible plant oils [13].

The aim of this review is to provide a state-of-the-art of the technological innovations for the extraction of edible vegetable oils and their supplementation with phenolic compounds, focusing on two main aspects: (i) emerging technologies aimed at improving the extraction yield as well as the nutritional, organoleptic and functional properties of oils and (ii) stabilisation approaches designed to enrich and fortify edible vegetable oils with phenolics. In addition, special attention is given to the combination of extraction and stabilisation techniques for developing bioactive ingredients and functional foods based on vegetable oils.

## 2. Materials and Methods

This systematic review was conducted according to the Preferred Reporting Items for Systematic Reviews and Meta-Analyses (PRISMA) guidelines 2020 [12], focused on the search on the electronic databases Web of Science, PubMed and Scopus for the selection of papers recently published, which was extended over the last 10 years. Additionally, this review was registered in the PROSPERO system (International Prospective Register of Systematic Reviews) under the registration ID CRD42024535490. To select studies evaluating the emerging technologies and the stabilisation approaches, searches were performed using different combinations: “Vegetable oils” OR “Edible oils” AND “Conventional technologies” OR “Pulsed Electric Field” OR “Ultrasound” OR “High-pressure processing” OR “Homogenizer” OR “subcritical water extraction” OR “enzymatic assisted extraction” AND “Bioactive compound” OR “phenol” OR “phenolic”; “Vegetable oils” OR “edible oils OR olive oil” AND extraction; “Bioactive compound” AND “emulsion” AND “water in oil” AND “nanoemulsion” OR “nano-emulsion”, “water in oil emulsion*” OR “W/O emulsion”. Duplicate articles from the consulted databases were removed. The inclusion criteria were applied through a two-stage process. Initially, the titles and abstracts of the manuscripts were assessed, and those that met the criteria were selected for a more comprehensive review of the full texts. The selected studies were required to focus on technologies related to vegetable oils and investigate phenolics formulated as water-in-oil (W/O) emulsions, microemulsions, or nanoemulsions. Finally, a language filter was applied, limiting the selection to those written in English.

**Figure 1:**
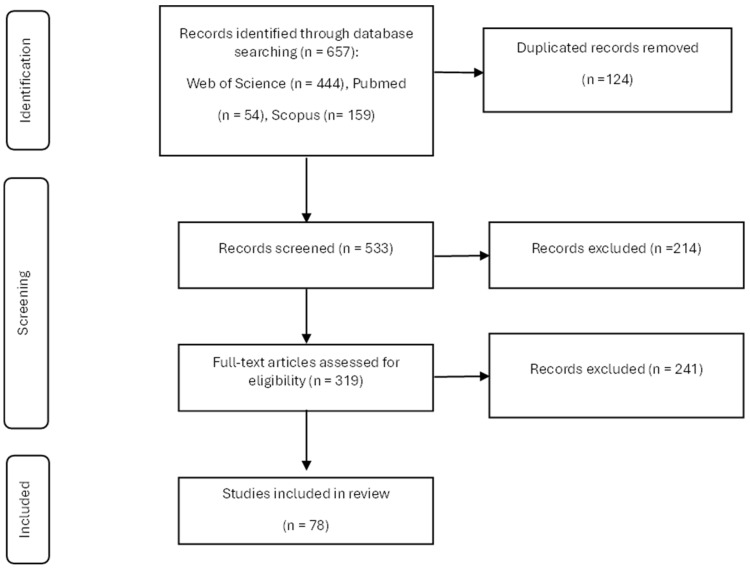
Study selection process for the systematic review.

A comprehensive literature search yielded a total of 657 articles. Following the removal of duplicates, 464 articles underwent a review process, during which 214 were discarded upon examination of the abstracts. Applying the full-text inclusion criteria resulted in the exclusion of 241 articles, yielding a final selection of 78 studies that met all the criteria and were included in the systematic review (Figure 1). Each study was assessed independently by the reviewers.

## 3. Results

### 3.1. Trends in Edible Oil Extraction Technologies

In recent decades, diverse innovative and advanced technologies have been successfully integrated into the plant oil industry, leading to substantial improvements in the efficiency, effectiveness and optimisation of oil extraction processes. These technologies have been developed to extract oils from a diverse range of vegetable matrices and sources, resulting in enhanced yield, quality and sustainability. Moreover, these innovations have not only enhanced the rate and yield of the extraction techniques but also reduced their environmental impact and resource consumption, thereby rendering them more economically viable and ecologically responsible. These techniques include pulse electric fields (PEF), high hydrostatic pressure (HHP), ultrasound-assisted extraction (UAE) and other technologies such as enzyme-assisted extraction (EAE) and subcritical water extraction (SWE).

#### 3.1.1. PEF

PEF is a non-thermal technology that can be used as a pretreatment before the extraction of edible oils from oil-bearing food matrices in the industry. This technique relies on the application of high-intensity electric fields in the form of exponential decay or squared pulses of short duration. Such pulses can degrade plant cells by electroporation of the cell walls, increasing their permeability. This results in a significant increase in the oil yield with shorter extraction times and lower energy consumption [10]. Although PEF technology is considered a non-thermal technology, it can generate short-term temporary temperature spikes that do not negatively impact the final product’s sensory and nutritional quality.

The potential of PEF-assisted extraction is based on several technical parameters such as electric field strength, pulse type (i.e., exponential decay, square, monopolar and bipolar), frequency and peak duration, relaxation time between pulses and applied energy.

Numerous comparative studies have investigated the application of PEF technology in producing edible oils. Table 1 summarises the main PEF technical parameters and their effects on oil quality. Concerning EVOO, PEF has been applied in different olive cultivars. EVOO from Manzanilla, Hojiblanca [11], Empeltre [15], Tsounati, Amfissis Manaki [16], Tsounati (Sparti, Greece) [17], Carolea, Coratina, Ottobratica [18], Picholine [19], Koroneiki [20] and Nocellara del Belice [21] olive varieties produced by conventional extraction process were compared with those obtained using PEF. The conventional process includes several unit operations such as milling and malaxation (i.e., slow mixing of the milled olives) with a gas controller system, two-phase separation with a decanter and a final polishing step where a vertical centrifuge removes residual water. PEF extraction systems are equipped with a pulse generator connected to a PEF treatment chamber, coupled to the process line, where electric pulses are applied to the food product. PEF treatments are applied before milling [16] (i.e., directly onto the whole olive drupes) as well as before [19,20,21] or after [11,15] the malaxation step. The electric field was set around 2 kV·cm^−1^ in all cases, while the delivered specific energies ranged between 1.6 and 70 kJ·kg^−1^, depending on the study. Overall, EVOO produced using PEF-assisted extraction showed higher oil yields for all cultivars treated and all process configurations. This indicates that the olive tissues were efficiently disrupted, improving the oil extraction yield compared to the conventional mechanical procedure.

The PEF-assisted experiments revealed a possible cultivar-dependent effect on oil extractability caused by the different fruit flesh/pit ratios and the moisture and oil contents of the olives when PEF was applied before or after the malaxation step. These factors can affect the transmembrane potential of fruit cells by reducing or increasing the extent of cell disruption. When PEF is applied to the raw drupes before milling, the effectiveness of the technology was significantly affected by the olive drupe dimensions [16]. Therefore, the operating conditions for PEF treatment must be optimised according to the olive variety and system configuration to achieve an economically feasible process that allows high extraction yields while limiting the costs associated with energy consumption.

Regarding the quality parameters of these oils, the free acidity (FA), peroxide value (PV) and spectrophotometric indices were comparable to those of conventionally extracted EVOO for most varieties. Only the Empeltre variety presented a significantly higher PV [15], whereas Tsounati, Amfissis and Manaki cultivars showed a slight increase in their FA values compared to conventionally extracted oils [16], although always remaining below the maximum limits for EVOO according to EU legislation. Oil quality indicators such as saponification value, K_232_, K_270_ and ∆K also showed no significant changes in the Koroneiki variety [20]. Concerning oxidative stability, studies that evaluated this parameter using the Rancimat accelerated oxidation test showed that PEF-assisted extraction did not affect the oxidative induction times of the produced oils or slightly increased them, indicating an improvement in oxidative stability. In some PEF chamber configurations, the electrodes are in direct contact with the olive paste so that ion metals such as iron or copper can be transferred to the food matrix and act as catalysts of lipid oxidation processes. However, the reported data indicated a negligible effect of metal release from the electrodes or a counteracting effect of the antioxidants present in the olive paste.

The main observed effect of PEF treatment on EVOO characteristics, beyond the increase in the yield, was the increase in phenolic content, suggesting that PEF favours the release of phenolic compounds and their subsequent solubilisation in the oily phase. This effect was common in all studies, regardless of the technique used for the analysis (i.e., Folin–Ciocalteu assay, HPLC-DAD or HPLC-MS). However, genetic differences among olive cultivars strongly influence the extent of this increase in phenolic content. Indeed, the Carolea, Coratina and Ottobratica varieties underwent a significant increase in phenolic content [18] when PEF assisted the extraction, while a very slight effect was found for other olive cultivars such as Manzanilla, Empeltre, Picholine or Nocellara del Belice [11,15,19,21]. Total phenolics, flavonoids and oleuropein (OLE) were also increased by 7.6%, 18.3% and 76%, respectively, in the Koroneiki variety [20]. In another study focusing on the Tsounati variety, PEF-treated olive oil showed significant enrichment of phenolic compounds. Hydroxytyrosol (HT), the predominant phenolic component, showed a 68% increase compared with the control. Similarly, tyrosol levels were almost doubled in the PEF-treated samples (93.84 mg/kg) compared with the control (57.23 mg/kg). The total lignan content, consisting of pinoresinol and 1-acetoxypinoresinol, reached approximately 399 mg/kg in the PEF-treated oil, whereas the OLE concentration was 64% higher than that in untreated samples. Furthermore, the apigenin concentration in PEF-treated olive oil (16.3 mg/kg) was notably increased compared to untreated samples (7.9 mg/kg) [17]. The reported differences among cultivars are due to the intrinsic variation in the polyphenol glycoside content in the olive fruit and the activity of the endogenous glucosidase, polyphenol oxidase and peroxidase enzymes, which are responsible for the biosynthesis of the characteristic phenolic composition of olive oil.

The tocopherol content was also evaluated in the different EVOOs, and it was observed that the PEF treatment affected the concentration of α-, β-, γ- and δ-tocopherols only in the Koroneiki variety [20]. This different behaviour, in contrast to the effect on the phenolic composition, has been attributed to the lipophilic nature of tocopherols, which means that these components are completely solubilised in the oil during the milling step; thus, PEF could not enhance this solubilisation as happened with phenols. As a result, only genetic and agronomic factors determine their final concentration in the EVOO, independent of the extraction process [11].

Regarding volatile composition, control and PEF-treated EVOO did not show significant differences in most cultivars, indicating that the treatment did not alter the organoleptic properties. However, Manzanilla EVOO showed an increase in (*E*)-hex-2-enal content when PEF was applied, although sensory evaluation by a panel test did not reveal any defects or off-flavours [11]. On the other hand, Nocellara del Belice EVOO showed a decrease in alcohol content compared to the control, which is associated with fruity and ripe fruit attributes [21]. In both cases, these changes in volatile components are related to a possible effect of the PEF technology on the lipoxygenase pathway.

From an industrial scale-up perspective, electric pulse technologies are emerging as promising alternatives that offer significant advantages in improving the efficiency of EVOO production while maintaining quality standards. The study conducted by Dias et al. (2024) [22] compared PEF-assisted olive oil extraction from the Galega Vulgar cultivar (30 min of malaxation) with the traditional method (45 min). PEF reduced malaxation time by 33% without affecting yield or extra-virgin quality. Regarding oxidative stability, the olive oil produced with PEF technology showed a slightly shorter oxidation induction time than the control sample. PEF treatment had no significant effect on the total phenolic content, slightly reducing it by 3.7%. Similarly, no statistically significant differences were observed in the tocopherol concentrations. Concerning the organoleptic properties, both the control and the PEF olive oils were considered EVOO.

Tamborrino et al. (2022) [23] also investigated the application of PEF technology in a pilot-scale industrial olive oil extraction process. The treatment of olive paste from the Coratina cultivar with PEF at a specific energy of 5.1 kJ/kg significantly improved oil extractability without compromising legal quality standards or sensory characteristics. In addition, PEF treatment increased the content of phenolic compounds, particularly OLE derivatives, which improved the antioxidant activity and enhanced the oil’s bitter and pungent sensory notes, contributing to its health benefits and overall quality. The application of PEF technology has also been studied in Picholine olive paste under actual large-scale extraction conditions. When used as a pretreatment prior to oil separation, PEF effectively improved both the extractability and concentration of bioactive compounds, particularly increasing the total phenolic content (TPC), with OLE showing a notable improvement. Importantly, using PEF did not significantly alter sensory attributes, quality parameters, or α-tocopherol levels, which is consistent with the findings of similar studies [19]. In the Arróniz olive oil variety, applying PEF treatment to the olive paste resulted in a 13.3% increase in extraction yield compared to the untreated control, without any negative impact on the oil’s chemical composition or sensory characteristics. In addition, the treatment resulted in a significant increase in total phenolics, phytosterols and tocopherols, highlighting this technology’s potential to improve yield and nutritional value [24].

The application of PEF technology in the extraction of edible oils extends beyond olive oil, demonstrating its versatility and potential for enhancing both yield and quality. Negi et al. (2023) [25] reported yields of up to 90% in virgin coconut oil extraction using PEF, attributing this efficiency to electrical disruption of cell membranes and enhanced permeability through electroporation. Furthermore, PEF-extracted coconut oil exhibited the highest TPC compared to other methods, such as microwave heating, ohmic heating and ultrasonication. In rapeseed oil extraction, PEF application increased TPC and oxidative stability, although higher electric field strengths led to a reduced extraction efficiency [26]. For sunflower oil, PEF resulted in a 2.3% increase in yield in a pilot-scale study, with slight modifications in acidity and peroxide index that did not compromise oil quality. TPC also increased significantly [27].

In accordance with the results reported in this section, when applied to the production of EVOO, PEF increases yield, phenolic content and antioxidant properties without compromising sensory or nutritional qualities. The effectiveness of a plant depends on several factors, such as cultivar and process parameters, which must be optimised for industrial scalability. PEF also shows promise in other oils such as coconut, rapeseed, sunflower and peony, consistently increasing yield and bioactive content, making it an innovative solution for the edible oil industry.

#### 3.1.2. HHP

HHP is a non-thermal technology in which food is exposed to very high hydrostatic pressure in the range of 100–800 MPa. This technology has been used primarily to preserve food against spoilage because it can inactivate microorganisms and limit chemical reactions, thus maintaining or even improving food quality [28]. Furthermore, HHP can alter the textural properties of foods. For example, it promotes the separation of meat from shellfish shells by altering the quaternary and tertiary protein structures [29]. It also disrupts cell integrity, increases cell membrane permeability and allows for the free movement of water and metabolites, thus softening plant tissues [30]. In this regard, HHP is a safe and effective technology for increasing the yield of oil extracted from plant sources while preserving nutritional properties. The main technical parameters controlling HHP performance are the applied pressure and the duration of the HHP treatment.

Several studies have evaluated HHP application to produce EVOO. Table 2 shows HHP parameters and processing conditions and their effects on oil quality. Arbequina olive fruits were subjected to HHP to extract EVOO, evaluating specific parameters such as pressure levels of 300 and 600 MPa and treatment durations of 3 and 6 min [31]. The quality analysis revealed a significant increase in the PV due to the application of HHP, highlighting an increase in the primary oxidation of lipids. In this sense, the initiation of lipid oxidation was primarily attributed to membrane damage induced by HHP, leading to the release of radicals or radical precursors [32]. Consequently, the oxidative stability at 300 MPa was lower than that of the control EVOO prepared without HHP. Surprisingly, EVOO extracted by HHP technology at 600 MPa did not show any change in its oxidative stability with respect to the control, which was attributed to the release of antioxidant components not evaluated in this study or to the formation of new ones. Regarding phenolic content, HHP treatment led to a reduction in phenolic compounds. Consequently, the protective effect against primary oxidation observed in EVOO produced at 600 MPa is likely attributable to antioxidant compounds other than polyphenols. Regarding other minor components, HHP increased the content of pigments such as chlorophylls and carotenoids, resulting in greener EVOOs, whereas the content of α-tocopherol and squalene was not altered.

HHP treatment was also evaluated for the production of EVOO from the Tsounati, Amfissis and Manaki cultivars at pressures of 200 and 600 MPa and treatment times of 1 and 5 min [16]. Contrary to previously reported results, the phenolic content and the different EVOOs’ oxidative stability slightly increased after HHP treatment, whereas the quality parameters were not significantly affected. This difference in behaviour may be attributed to the genetic differences between the cultivars, which determine their content of antioxidant compounds. In this regard, Tsounati EVOO showed the best results in terms of oxidative stability due to the higher occurrence of phenolic compounds in this variety. On the other hand, it should be highlighted that although cell disruption by HHP can cause the release of a higher concentration of phenolic compounds, these released phenols are more susceptible to enzymatic degradation; therefore, as mentioned in the PEF section, the diverse activity of endogenous enzymes of the different cultivars may play a crucial role in the phenolic content of the produced oil and, consequently, in its oxidative stability.

A study by Andreou et al. (2022) [17] also used olives of the Tsounati variety from Sparti, Greece to investigate the effects of HHP on olive oil yield, quality and oxidative stability. The olives were processed under optimal HHP conditions (600 MPa, 5 min; malaxation at 26 °C for 35 min) and compared with those obtained using conventional methods (no pretreatment, malaxation at 30 °C for 45 min). The results showed that HHP treatment increased olive oil yield and oxidative stability, attributed to the enhanced extraction of polyphenols and α-tocopherols. Further analysis of bioactive compounds revealed that HHP-treated olive oil had a 44% higher HT content than the control. The total concentration of lignans, including pinoresinol and 1-acetoxypinoresinol, was also significantly increased in HHP-treated samples. In addition, OLE content significantly increased, and the level of apigenin was higher in HHP-treated olive oil than in untreated samples. These results highlight the effectiveness of HHP treatment in improving olive oil’s oxidative stability and bioactive compound profile.

Other authors [33] applied HHP at a high intensity (600 MPa) to a mixed batch of olive fruit cultivars, including Frantoio and Moraiolo (proportions unknown) and evaluated different quality indices immediately after treatment and after 6 months. The HHP treatment did not cause a significant increase in oxidative indices such as PV, K_232_, or K_268_ after 6 months of storage, nor was there a significant difference in FA compared with the control. These results suggest that HHP treatment does not negatively affect the oxidative stability of olive oil during storage. In addition, the biophenol concentration remained elevated in the HHP-treated samples, with significant increases in decarboxymethyl ligstroside aglycone dialdehyde and its oxidised form. Although most other biophenols decreased over time, HHP treatment helped to maintain or increase certain compounds, such as ferulic acid and decarboxymethyl oleuropein aglycone. The levels of oleuropein aglycone and pinoresinol slightly decreased but remained relatively stable, indicating that HHP treatment may help to preserve and even enhance certain bioactive compounds in olive oil during storage. In conclusion, HHP treatment effectively preserved oxidative stability and increased the concentration of key bioactive compounds in olive oil during storage without negatively affecting its quality.

The scientific literature reviewed for HHP indicates that this technique is an effective non-thermal technology for improving olive oil yield, oxidative stability and bioactive compound content. Studies have shown that HHP-treated olive oil also increases levels of key antioxidants such as HT and lignans while maintaining or slightly improving oxidative stability during storage. The treatment also increases the presence of beneficial pigments such as chlorophylls and carotenoids without compromising other quality parameters. In addition, genetic differences among olive cultivars influence the effects of HHP on phenolic content and oxidative stability, highlighting its potential for tailored applications.

#### 3.1.3. UAE

UAE technology is based on generating ultrasonic waves that create cavitation—the formation and subsequent collapse of gas bubbles in a liquid medium. This process disrupts plant cell membranes, promoting the release of oil under mild processing conditions. The key parameters influencing UAE performance include energy output, frequency and intensity. UAE treatment increases the yield of oil extraction while preserving its quality [34]. In olive oil production, applying ultrasonic waves to olive paste can reduce preheating time through two mechanisms: thermal and mechanical effects. When the kinetic energy of ultrasonic waves is absorbed by a material, a thermal effect occurs. The formation, expansion and implosion of gas bubbles under high negative pressure enhance the release of soluble chemicals from plant tissues by breaking cell walls and facilitating mass transfer in olive tissues.

UAE has been applied to improve the efficiency of the oil extraction process. Table 3 summarises the application of this technique for edible oil extraction, highlighting key parameters, processing conditions and its impact on yield, efficiency and quality. Regarding EVOO, UAE has been applied to different olive cultivars such as the Coratina and Peranzana cultivars [35]. In this study, the ultrasonic treatment of olive paste before malaxation reduced this phase without altering the main quality parameters, including FA, PV and spectrophotometric indices. In terms of chemical composition, ultrasound treatment increased the levels of tocopherols, carotenoids and chlorophylls and reduced phenolic content was attributed to the action of oxygen from gas bubbles on these compounds together with the activation of endogenous oxidoreductase enzymes caused by ultrasound waves, which could promote the oxidation of polyphenols. Because of this decrease in phenolic compounds, the sensory profile of the EVOO changed with a reduction of bitter and pungent notes, although the fruity notes were unaffected.

As a way to counteract the potential detrimental effect of oxygen on olive oil quality, Iqdiam et al. (2019) evaluated the combination of UAE and oxygen concentration control in the malaxation headspace to produce EVOO from the Arbequina cultivar [36]. In this case, the comparison between control and ultrasound-treated oils under atmospheric O₂ concentration during malaxation showed no effect on quality parameters while increasing the content of tocopherols, carotenoids and chlorophyll, which was consistent with a previous study. However, the reduction in the oxygen concentration during the malaxation phase increased the oxidative stability of EVOO, accompanied by a subsequent reduction in the PV. A lower FA was also obtained with lower oxygen percentages, whereas the spectrophotometric indices were not affected. This decrease in oxygen levels also contributed to improving functional parameters, including the levels of phenolic compounds and tocopherols. The increase in these phytochemicals affected the organoleptic qualities of EVOO, leading to an increase in pungent and bitter notes, as well as in the overall fruit attributes. Therefore, controlling the oxygen content in the malaxation headspace counteracts ultrasound’s detrimental effect on the polyphenol content, thereby improving the overall quality of the produced EVOO.

Clodoveo et al. (2017) [37] designed a full-scale sono-exchanger for the virgin olive oil industry to be placed between the crusher and the malaxer. In this study, the EVOO from the Coratina cultivar produced with the designed configuration maintained quality parameters comparable with those of traditionally produced EVOO. In contrast, the tocopherol, carotenoid and phenolic contents increased. This improvement in phenolic composition was mediated by the inhibitory effect of ultrasound treatment on polyphenol oxidase, indicating the crucial importance of proper optimisation of UAE parameters to avoid deterioration of the quality of the produced oils.

Other edible vegetable oils have also been extracted using UAE technology. In this sense, UAE significantly enhanced the yields of grape-seed oil and phenolic compound extraction compared to conventional methods. The process preserved the fatty acid composition and produced oils with acidities below the FAO/WHO limits, indicating minimal degradation. However, sonicated oils showed higher PV after 30 d of storage, exceeding recommended limits, highlighting the need for preservation strategies such as refrigeration and antioxidants. The improvement of phenolic content and antioxidant capacity observed in sonicated samples were attributed to ultrasound-induced cell wall disruption, which facilitated the release of bioactive compounds [42]. Oil extraction from *Cnidoscolus quercifolius* (Favela) seeds was also investigated using the UAE method, using ethanol as solvent. This unconventional approach effectively improves oil recovery while preserving bioactive compounds and enhances the antioxidant and nutritional qualities of the oil. The optimised process yielded an oil rich in bioactive compounds, such as tocopherols and β-sitosterol, which were strongly correlated with the antioxidant capacity. In addition, the oil had a high content of unsaturated fatty acids, mainly linoleic and oleic acids. The extraction performance was comparable to conventional Soxhlet methods, with similar yields but improved oil quality and antioxidant properties [41].

Loizzo et al. (2019) [39] reported that the use of UAE to obtain seed oils from two varieties of *Opuntia ficus-indica* (Sanguigna and Surfarina) was less effective than the conventional Soxhlet method in terms of performance and bioactive compound recovery. This method preserved the original fatty acid composition, rich in polyunsaturated fatty acids (PUFAs), especially linoleic and oleic acids. However, UAE resulted in lower tocopherol and carotenoid contents than Soxhlet extraction. The antioxidant activity and enzyme inhibitory effects of the UAE-extracted oils were moderate, indicating that this method, while effective for oil recovery, resulted in lower enrichment of bioactive compounds.

Nie et al. (2020) [38] compared the physicochemical properties of *Paeonia lactiflora* Pall seed oils obtained by UAE, hot-pressing and supercritical fluid extraction (SFE). UAE produced oils with moderate acidity and superior oxidative stability compared to hot-pressing, indicated by lower PV. Although SFE had the highest oxidative stability, its oil yield was lower than that of UAE, although it achieved higher transfer of tocopherols and phytosterols to the extracted oil. Pressing produces the lowest oil yields and the highest PV. In addition, UAE effectively extracted bioactive phenolic compounds, yielding comparable or higher concentrations of flavonoids, hydroxybenzoic and hydroxycinnamic acids compared to hot-pressing.

UAE demonstrated significant advantages over traditional solvent extraction for improving the composition and quality of cactus fruit seed oil (CFSO) [40]. UAE effectively increased the concentrations of lipid concomitants, including linolenic acid, α-tocopherol and canolol, while maintaining the thermal stability of the oil, as evidenced by its unchanged crystallisation and melting behaviour. In addition, UAE significantly affected the CFSO phenolic acid and tocopherol content. It was concluded that increasing ultrasound intensity (150, 400 and 600 W) led to higher levels of key bioactive compounds, with the highest concentrations of canolol and sinapic acid reported at 600 W. In contrast, syringic and salicylic acids remained relatively unaffected. UAE also significantly increased α-tocopherol and γ-tocopherol concentrations, with the peak values achieved at the highest ultrasound intensity. However, excessive ultrasound power increases the risk of oil oxidation and free radical formation. These results highlight UAE as an effective technique for optimising the CFSO’s nutritional and functional properties while preserving its structural and thermal integrity.

Overall, the results provided by these studies pointed out that UAE improves the yield and quality of edible oils by increasing bioactive compounds such as tocopherols and phenolic acids while maintaining their structural and thermal properties. The proposed method outperforms traditional methods but requires careful optimisation to avoid oxidative degradation and offers a sustainable solution for producing high-quality oils.

#### 3.1.4. EAE

EAE is an environmentally friendly technology that uses food-grade enzymes to break down plant cell walls, allowing for the release of intracellular components such as oils and bioactive compounds. The cell walls of oleaginous plants have a similar composition consisting of cellulose, hemicellulose, proteins and pectins. Consequently, enzymatic-assisted extraction of oil from these plants employs single enzymes or enzyme blends, including cellulases, hemicellulases, pectinases and proteases [43]. This method has emerged as a particularly advantageous approach for extracting biomolecules from plant matrices due to its use of safer solvents, enhanced oil quality and reduced environmental impact by decreasing emissions of volatile organic compounds. EAE is recognised for its adherence to the principles of green chemistry, offering a sustainable alternative to traditional solvent-based extraction methods. The process entails the optimisation of technical parameters, such as the sample-to-water ratio, pH, temperature, duration and enzyme concentration, to maximise efficiency and yield [44,45,46]. Table 4 provides an overview of the technical parameters used in EAE of edible oils, focusing on key processing conditions and their effects on yield, efficiency and quality. While this method has been successfully applied to extract edible oils from various vegetable matrices, its use in olive oil extraction remains undocumented because it has primarily been employed for oil extraction from seeds and nuts.

Polmann et al. (2019) [47] investigated the EAE of pecan nut oil and compared it with mechanical pressing (MP). The study evaluated the physical–chemical properties and chemical composition of the oils obtained by both methods. Initially, a Plackett–Burman design was used, followed by a central composite rotatable design, which optimised the extraction conditions. The highest yield of EAE with Alcalase^®^ (1.50 g/100 g) was achieved after 4 h at pH 8.00, 52 °C, 0.40 g/mL substrate concentration and 120 rpm agitation. Under these conditions, EAE produced an 8.1% higher yield than MP.

The fatty acid compositions of the oils were similar, except for oleic and linoleic acids. Therefore, EAE yielded higher oleic acid and lower linoleic acid levels. Furthermore, EAE resulted in a lower total tocopherol content, particularly a reduced γ-tocopherol concentration. Subsequent analysis of peroxide, anisidine and acidity values revealed no significant differences in oil quality between both methods. Furthermore, the Rancimat method, which measures oxidative stability over 12 h, revealed strong resistance to oxidation in oils from both techniques, with no significant differences observed. Regardless of the differences in fatty acid profiles and tocopherol contents, the oils extracted by both methods exhibited comparable oxidative stability and high quality.

Another relevant study on the use of EAE was conducted by Jiang et al. (2020) [48], who compared the effects of three extraction methods—cold-pressing (CP), hot-pressing (HP) and EAE—on the trace components of peanut oil. The peanut kernels were washed, heated at 60 °C and then ground into a fine pulp prior to EAE. Subsequently, the alcalase enzyme (1.5% of the paste weight) was incorporated at pH 8.5 and the mixture was subjected to reaction at 60 °C for 3 h. A subsequent analysis of the peanut oil extracted using these three methods via liquid chromatography–mass spectrometry (LC-MS) revealed substantial variations in the composition of the trace active components. The EAE method resulted in the highest levels of wogonin and was the only method that produced methyl 4-hydroxybenzoate. However, for other compounds, such as isovanillin, EAE did not outperform HP or CP, with the lowest concentration of isovanillin being found in EAE oil. These results suggest that the enzymatic extraction process significantly influences the composition of the trace active components of peanut oil.

EAE methodologies have also been employed in the context of seed oil extraction to enhance yields and optimise oil quality. In this regard, Ribeiro et al. (2016) [49] conducted a study to evaluate the impact of EAE on sesame oil extraction. A 2^3^ factorial design was employed to assess the influence of the raw material-to-water ratio (1:6 to 1:10), extraction time (4 to 8 h) and enzyme concentration (6 to 10%, for both pectinase and alcalase) on oil yield. Following the optimisation of the yield, an array of additional parameters was compared with conventional methods (CP and solvent extraction), including the fatty acid profile, antioxidant activity 2,2-diphenyl-1-picrylhydrazyl (DPPH) and L-ORAC and phytosterol and tocopherol content. The findings indicated that EAE yielded a significantly lower amount of oil than conventional methods. However, EAE exhibited a notable improvement in the fatty acid composition, particularly an increase in polyunsaturated PUFA and omega-6 content. Furthermore, EAE enhanced phytosterol levels, particularly sitosterol and campesterol, while preserving γ-tocopherol content. Notably, the antioxidant activity, EAE oil exhibited twice the antioxidant capacity, compared with traditional methods These results indicate that EAE is more efficient for extracting bioactive compounds with antioxidant potential than conventional methods.

The use of EAE for sunflower seed oil was investigated by de Aquino et al. (2019) [43]. Using a Box–Behnken design, the authors evaluated the effects of temperature (40–60 °C), enzyme concentration (1–9% *v*/*v*) and seed-to-water ratio (1:5–1:15 g/g) on oil yield. The impact of a buffered medium and the use of the surfactant Tween 80 for oil recovery was also assessed, with results compared to Soxhlet extraction in terms of fatty acids, phytosterols and tocopherols. The optimal conditions were a seed-to-water ratio of 1:5, pH 4.5, 1% Celluclast^®^ 1.5 L and 60 °C. The pH value was optimised to achieve maximal enzyme activity and minimise emulsion formation. Contrary to expectations, a higher enzyme concentration results in a lower oil extraction yield. This could be explained by the enhanced release of compounds that contribute to the formation of stable emulsions, thereby limiting the recovery of free oil. While fatty acid profiles from the EAE and Soxhlet methods were comparable, linolenic acid levels were lower in the EAE-extracted oil. This discrepancy may be attributed to the potential degradation of linolenic acid in the aqueous medium during EAE, given the high susceptibility of polyunsaturated fatty acids to oxidation. The EAE process demonstrated superior phytosterol content, potentially due to enzymatic activity that disrupts cell walls, promoting phytosterol release. Furthermore, the tocopherol levels obtained were comparable to those achieved through conventional Soxhlet extraction.

In line with previous studies, Arrey et al. (2022) [50] investigated EAE for oil recovery from njangsa seeds (*Ricinodendron heudelotti*), comparing it with hexane- and water-based extractions. The seeds were homogenised with water at a 1:6 ratio, and a 2% enzyme dosage (based on flour weight) was applied using hemicellulase, protease, pectinase and amylase under optimal activity conditions (Table 4). While hexane extraction produced higher yields, EAE significantly outperformed the water-based control, suggesting that enzymes enhance oil extraction. Notably, EAE utilising hemicellulase achieved the highest oil recovery among all the enzymes tested. All the extraction methods yielded oils with comparable fatty acid profiles, with α-eleostearic acid as the predominant component. However, volatile compound analysis revealed that hexane extraction produced more hydrocarbons, while EAE-hemicellulase oil was richer in alcohols, aldehydes and esters, demonstrating the influence of the extraction method on the chemical composition. The EAE oils demonstrated superior quality, exhibiting significantly lower oxidation levels, as indicated by reduced levels of free fatty acids (FFA), PV, AV and p-AV, suggesting enhanced oxidative stability.

Zhang et al. (2023) [54] evaluated the impact of 12 processing technologies on high-oleic rapeseed oil (HORO), combining pretreatments (microwave (M) and roasting (R)) with extraction methods such as CP, hexane extraction (HE), subcritical butane extraction (SBE) and EAE. Microwave pretreatment was applied at a frequency of 2450 MHz and 800 W power for 7 min, while roasting consisted of heating at 140 °C for 1 h. EAE was carried out by mixing the pulp with boiling water (1:7, *w*/*v*), adding a mixture of polysaccharide enzymes (3% *w*/*w*) and incubating at 48 °C for 5 h. Finally, the pH of the slurry was adjusted to 9, and 1.5% (*v*/*v*) alcalase was added and stirred for 2 h at 60 °C.

EAE achieved the highest oil yields (56.4–59.7%), effectively breaking down the rapeseed cell walls, although pretreatment reduced yields compared to EAE without pretreatment. In terms of bioactive compounds, M-EAE retained significantly higher levels of α-tocopherol compared to most methods, enhancing antioxidant capacity. The highest levels of polyphenols were observed in the CP treatment, followed by M-HE and EAE. On the other hand, the lowest polyphenol content was found in R-SBE, which was significantly lower compared to all other treatments. Significant differences were also noted between M-SBE and R-EAE, with M-SBE displaying a substantially reduced polyphenol concentration. Single EAE treatment, as well as the combined M-EAE and R-EAE led to high recovery rates of sterol compounds, particularly campesterol and sitosterol.

The AV and PV values revealed significant differences across methods, with EAE showing significantly higher AV values, particularly with the combined R-EAE treatment, indicating more FFA. The combination M-EAE demonstrated the lowest peroxide values, indicating superior oxidative stability, while CP exhibited the highest PV, reflecting lower stability. EAE oils also showed improved thermal stability and low rancidity, evidenced by the lowest iodine values.

Overall, these authors demonstrate that EAE is an effective extraction method for rapeseed oil extraction, with the combination M-EAE standing out for its antioxidant capacity, mainly due to higher α-tocopherol retention and superior oxidative stability. Both M-EAE and R-EAE offered favourable bioactive compound profiles, including polyphenols and phytosterols, although slightly inferior to those achieved by HE and M-HE. Despite this, EAE methods significantly improved oil quality, stability and bioactive content compared to traditional methods.

In industrial applications, mechanical pressing of dry seeds is often chosen as a standard method for oil extraction. The study by Thomsen et al. (2024) [55] demonstrated the feasibility of an enzyme-assisted method for extracting rapeseed oil with minimal water addition (1 mL per 100 g of seeds). The use of cellulolytic and pectolytic enzymes promotes carbohydrate breakdown, resulting in increased reducing-sugar content. Consequently, oil recovery during mechanical pressing was significantly enhanced, improving from 60% to 65%. In addition, the enzymatic treatment had no notable effect on oil quality, as determined by electron spin resonance (ESR) spectroscopy. ESR analysis revealed neither an increased formation of radicals nor a decrease in the antioxidant capacity of the oil because the enzyme-assisted extraction could be detected.

More specifically, Atsakou et al. (2023) [52] investigated the application of EAE for the production of pumpkin seed oil using a combination of pectinase from Aspergillus sp. 391 and Celluclast^®^ 1.5 L at a volumetric ratio of 1:1. The use of this enzyme combination resulted in a substantial enhancement of oil recovery compared with the use of individual enzymes or Soxhlet extraction. This enhancement can be attributed to the synergistic effects of the enzymes in disrupting the bonds between pectin and cellulose. Moreover, no significant differences in fatty acid composition were observed between the extraction methods, with linoleic acid (C18:2) being the most abundant in all cases. In terms of oil quality, EAE yielded the best results, as demonstrated by significantly lower FA and PV values compared to Soxhlet extraction. This difference can be ascribed to the milder thermal conditions associated with EAE (50 °C vs. 90 °C), which minimises peroxide degradation and better preserves oil quality.

A more recent study [51] also employed EAE with *Bacillus licheniformis* protease to evaluate its effectiveness in recovering oil from fruit seeds and kernels, including mango, lemon, pumpkin, papaya, peach and cherry seeds. This method demonstrated its potential for oil recovery, extracting 10–30% of the total oil content as free phase after centrifugation in substrates such as mango, lemon and pumpkin seeds. However, most of the oil remained in the residual pellet, requiring additional solvent extraction for full recovery. The study also found no significant differences between EAE and the combined acid/solvent extraction methods, which produced oils with similar fatty acid profiles, indicating that both methods effectively recover the same types of fatty acids while preserving nutritional quality. However, higher concentrations of unsaponifiable compounds, particularly β-sitosterol and squalene, were obtained in pumpkin seed oil through combined acid/solvent extraction. In terms of tocopherol content, the α-tocopherol levels in pumpkin seed oil extracted using EAE were slightly lower than those obtained through acid hydrolysis + Soxhlet extraction, while β-tocopherol was only identified in the oil extracted via EAE.

Other authors [56] have also aimed to investigate the impact of various enzymes, including single enzymes, chimeric enzymes and the modified CBM3-fused variants (i.e., carbohydrate binding module 3), on oil yield and fatty acid profile in coconut kernel oil extraction and compare it with aqueous extraction (AE). For this purpose, mature coconut kernels were ground in a phosphate buffer and specific enzymes were added. Under these experimental conditions, EAE achieved a significantly higher oil yield than AE. The use of enzymes such as the recombinant endo-mannanase (ManB-1601), cellobiose hydrolysases (CelB and CelBΔCBM) resulted in a 13% increase in oil yield, whereas the fusion of CBM3 with the complex mannanase–xylanase (ManB-XynB) achieved a 22% increase in oil yield. Confocal laser scanning microscopy revealed that EAE, particularly employing the combination ManB-XynB-CBM protein, resulted in significantly greater disruption of the coconut endosperm cell walls, releasing the oil droplets trapped within. Fatty acid methyl ester (FAME) analysis revealed that the fatty acid profiles of the oils extracted by AE and EAE were similar, composed mainly of lauric acid, myristic acid and palmitic acid, and both met the standards established for coconut oil.

To further enhance both the oil extraction yield and the quality of the oil, various extraction techniques have been combined with EAE. Ezeh et al. (2016) [53] explored the combination of high hydrostatic pressure and EAE on tiger nut oil extraction. The process involved a pretreatment at 300 MPa for 20 min, followed by EAE with a sample-to-water ratio of 1:4 and a 0.5% enzyme concentration (a mixture of amylase, alcalase and Celluclast^®^) for 6 h of incubation. This combined pretreatment led to significant improvements in tocopherol and phenolic content of the extracted oil, while preserving the FFA profile and PV.

Similarly, Moradi et al. (2018) [46] evaluated the combined effects of ultrasound (U) or PEF on the EAE of sunflower oil. The results indicated that the optimal conditions for the highest sunflower oil recovery during a 2 h EAE were a cellulase-to-pectinase ratio of 2:1, enzyme concentration of 2%, pH 4.5, liquid-to-solid ratio of 6:1 and temperature of 40 °C. Under these conditions, the combined treatment U-EAE (24 kHz, 250 W) and PEF-EAE (1.2 kV/cm, t PEF = 0.4 s) for 30 min significantly improved oil extraction yield. Tocopherol content in sunflower oil exhibited slight variations depending on the extraction method. Specifically, U-EAE led to a minor decrease in total tocopherols, primarily due to a reduction in α-tocopherol levels. In contrast, PEF-EAE caused a slight increase in α-tocopherol content, while no significant differences were observed in the levels of β- and γ-tocopherols between the methods. Regarding the physicochemical characteristics, sunflower oils extracted using EAE, U-EAE and PEF-EAE exhibited similar refractive index values at 40 °C, all within the standard range for sunflower oil, with no significant differences among them. The acidity value, an indicator of oil purity and edibility, showed no significant differences between EAE and U-EAE, while PEF-EAE slightly increased free acidity. Nevertheless, all values remained within acceptable limits for crude sunflower oil. Regarding the peroxide value index, no significant differences were observed between EAE and PEF-EAE. However, it was observed that the primary oxidation of the extracted oils was accelerated under the combined treatment U-EAE. Notably, PEF-EAE yielded the oil with the lowest PV, presenting the least rancidity. Nonetheless, all three techniques yielded PV below 20 mEq O₂/kg, adhering to the established standards for crude sunflower oil.

According to the literature reviewed, EAE proves to be an efficient, sustainable and environmentally friendly method for oil extraction from various plant seeds. It enhances oil yield, preserves oil quality and retains bioactive compounds, offering a promising alternative to traditional methods. EAE also reduces the need for harmful solvents, demonstrating its effectiveness and versatility across different plant materials while improving oxidative stability and antioxidant properties in extracted oils.

#### 3.1.5. SWE

SWE is an environmentally friendly method that uses water at temperatures above its boiling point (100–400 °C) and high-pressure conditions (typically > 5 MPa) to keep it in a liquid state. Subcritical water is significantly less polar than water at ambient temperatures, and its polarity decreases substantially with increasing temperature. Under these conditions, organic compounds such as lipids exhibit greater water solubility than conventional extraction methods. The combination of high levels of temperature and pressure enhances mass transfer, facilitating solvent penetration into the plant matrix and thereby improving extraction yields. From an operational perspective, key parameters influencing the SWE process include pressure, temperature and the sample-to-solvent ratio [57].

Table 5 includes the technical parameters used in the SWE of edible oils. SWE was used to extract sunflower oil by varying different parameters, including temperature (60–160 °C), extraction time (5–120 min) and raw material-to-solvent ratio (1/10, 1/20 and 1/30 g/mL) [58]. All experimental runs were conducted at a pressure of 30 bars, with extraction yields measured under different operational conditions. The optimal oil extraction yield of 44.3% was achieved at 130 °C and an extraction time of 30 min, using a sunflower-to-water ratio of 1/20 g/mL. Higher temperatures resulted in lower extraction yields, which was attributed to reduced disruption of cotyledon cells, where most of the seed oil is located. Additionally, temperatures above 130 °C caused a decrease in the water dielectric constant, which in turn reduced the solubilisation of the protein phase, where oil droplets are enclosed. The antioxidant activity of the lipid extracts, assessed by a photo-chemiluminescence method, increased with both temperature (60–130 °C) and extraction time (5–60 min). This increase was associated with the release of free soluble phenolic compounds (e.g., caffeic acid, ferulic acid) through hydrothermal reactions. The FFA content in the SWE-extracted oil was slightly higher than that in the control samples obtained by conventional Soxhlet extraction (4.4% *w*/*w* for SWE at 130 °C versus >3.5% *w*/*w* for Soxhlet-extracted oil). The increase in free acidity after SWE was expected due to the thermal degradation of triglycerides, which could be significantly minimised by operating at temperatures below 60 °C. Similarly, another study [59] evaluated the effect of SWE temperature on the FFA content in sunflower oil by varying temperature (130–240 °C) and extraction time (30–120 min). This study set the pressure and raw material-to-solvent ratio at 35 bar and 1/20 mg/L, respectively. The results showed that the temperatures tested in this study caused significant hydrolysis of triglycerides, compared to the previous research on sunflower oil. It was found that the FFA content remained relatively stable at 130 °C and 160 °C (2.32–2.62% *w*/*w* after 120 min) but increased substantially above 190 °C. Treatment at 240 °C led to extensive triglyceride hydrolysis, significantly reducing the quality of the extracted sunflower oil, with FFA content exceeding 70% *w*/*w*.

In addition to sunflower oil, SWE has also been used for extracting palm oil [60], with various pressure levels (30, 40, 50 bar) and temperatures (120–180 °C) tested. In this study, the solvent-to-sample ratio (5:1 mL/g) and extraction time (60 min) were fixed and the effect of pressure and temperature on extraction yield and free acidity was evaluated. The results demonstrated that increasing pressure and temperature led to higher extraction yields. This was attributed to the reduction in water viscosity with temperature, which enhances mass transfer and solvent penetration into plant particles. Unlike the results reported for sunflower oil, no increase in FAA was observed with increasing pressure or temperature within the studied ranges. This is likely because all temperature values remained below 190 °C. While the pressure values used in this study were higher than those in the sunflower oil study, their effect on triglyceride hydrolysis was not significant compared to temperature.

In a study conducted by Wu et al. (2018) [61], the response surface methodology (RSM) was employed to optimise the extraction yield of *Camellia oleifera* seed oil using SWE. The key parameters investigated were temperature (110–150 °C), extraction time (20–40 min) and solvent-to-material ratio (5:1–15:1 mL/g), and the optimal values for these parameters are presented in Table 5. Additionally, the physicochemical properties and quality of the oil obtained under these optimal conditions were compared with those extracted using conventional methods, such as cold Soxhlet extraction (SE) and CP extraction. The results revealed that SWE, SE and CP oils showed a similar fatty acid profile with oleic acid as the predominant fatty acid. Moreover, SWE also exhibited a relatively greater preservation of α-linolenic acid compared to other methods. Physicochemical analysis showed that SWE oils exhibited significantly lower acidity and PV than CP oils, indicating reduced triglyceride oxidation and hydrolysis. Furthermore, SWE resulted in a twofold increase in the levels of unsaponifiable substances, including fatty alcohols, sterols and hydrocarbons. This finding aligns with the higher Trolox equivalent values observed for SWE-extracted oils compared to those obtained via CP. These data suggest that SWE effectively extracts and preserves bioactive compounds, consequently enhancing the antioxidant capacity of the resulting oils.

The scientific research reviewed indicates that SWE has shown promising results for extracting edible oils, offering good yields and improved preservation of bioactive compounds, especially at optimised temperatures and times. While it can lead to increased free fatty acid content at higher temperatures, it also enhances antioxidant activity and oil quality when carefully controlled. Compared to traditional methods, SWE provides higher efficiency and better preservation of certain compounds. Overall, SWE is a valuable technique for oil extraction, though precise control of extraction parameters is essential for optimal results.

### 3.2. Technologies for the Incorporation of Active Ingredients into Vegetable Oil

Emulsions are colloidal systems comprising two distinct phases, where one phase is dispersed as fine droplets into a continuous phase. The most common emulsions in the plant oil industry are water-in-oil (W/O) systems, where an aqueous phase is dispersed within an oil matrix. These systems are produced to enrich vegetable oils with polar compounds (water-soluble bioactive, e.g., polyphenols) and incorporate oil ingredients into different food matrices.

Emulsion systems are thermodynamically unstable since both phases tend to separate by coalescence or sedimentation and require the presence of emulsifiers for their stabilisation. These are amphiphilic compounds that adsorb at the interface of the oil or water droplets, providing stability to the dispersed system by steric hindrance or electrostatic repulsion [62]. These stabilisers are incorporated into the emulsion to formulate micro- and nanoemulsions with reduced droplet size, which prevents emulsion destabilisation due to the lower tendency of submicron droplets towards coalescence. Due to their enhanced stability, both systems (micro- and nanoemulsions) are receiving much attention in the food industry.

Microemulsions are stable systems with average droplet sizes below 100 nm, generally produced under mild stirring at room temperature. In contrast, nanoemulsions are metastable systems with an average droplet size below 200 nm, which present a high interfacial area per unit volume. Their formulation requires higher energy inputs from high-speed mixing, sonication, or high-pressure homogenisation [63,64]. The application of ultrasound and HPH has addressed this stabilisation.

Emulsions are effective delivery systems for hydrophilic and lipophilic bioactive compounds, offering efficient encapsulation, enhanced stability and controlled release [65,66]. Among these, phenolic compounds stand out due to their functional and nutritional properties. Their potent antioxidant activity not only protects vegetable oils from peroxide oxidation, thereby extending their shelf life, but also contributes to health benefits, including anti-diabetic, anti-inflammatory, anti-carcinogenic and neuroprotective effects, as well as cardiovascular protection [67]. Several factors, such as the concentration of phenolic compounds, emulsifying agents and the type and amount of oil, influence the incorporation of phenolic compounds into vegetable oils via emulsification. Understanding how these variables affect emulsion stability is critical for optimising these systems’ functional and physical properties. This review section focuses on the second aim of applying W/O emulsions as carrier systems, highlighting the current advances in stabilising phenolic compounds in vegetable oils.

Table 6 shows the emulsification procedures used for encapsulating phenolic compounds in water-in-oil emulsions, highlighting key techniques, processing parameters and their impact on stability and efficiency. In the work by Fregapane et al. (2022) [68], virgin walnut oil (VWO), virgin pistachio oil (VPO) and refined olive oil (ROO) were enriched with phenolic-rich extracts from pistachio (5.1% wt.) and walnut (27.4% wt.) Both extracts were incorporated into the oil matrix using water-in-oil (W/O) emulsion and microemulsion systems to test different emulsifiers. A stable emulsion was obtained by dispersing 2% wt. water in the oil matrix by an ultrasound probe, adding 0.5% wt. polyglycerol polyrricinoleate (PGPR) as an emulsifier. This system presented a whitish opaque appearance, although it was remarkably stable in time. By contrast, 2% wt. of the emulsifier mixture of 3:2 lecithin-distilled monoglycerides (DMG) and 1% wt. of water allowed for obtaining VWO and ROO transparent and stable microemulsions.

The TPC content of the W/O emulsions ranged from 257 to 835 mg/kg and the TPC values of the microemulsions were between 238 and 499 mg/kg. Although the TPC values were lower under microemulsion conditions, these values were significantly higher than those of vegetable oils without extracts. Furthermore, it should be noted that these conditions gave rise to a transparent emulsion, a key factor for commercialisation. Fortification with phenolic extracts significantly improved the plant oils’ antioxidant activity, evaluated by the DPPH assay. For instance, the antioxidant capacity of VWO improved from 0.09 mmol Trolox/kg oil to 2.89 mmol Trolox/kg after fortification with walnut phenolic extracts and obtaining a microemulsion. Moreover, the oxidative stability of VWO, VPO and ROO emulsions and microemulsions was evaluated under accelerated testing conditions, employing Rancimat equipment and performing an oven test to determine the number of days required for the oil to achieve a PV of 15 mEq O_2_/kg, which is the upper value allowed for commercial products. In this regard, walnut and peanut extracts increased the oxidative stability of the emulsions and microemulsions prepared with VWO, delaying the time to reach the peroxide limit from 11.3 to 17.9 and 19.7 mEq O_2_/kg, respectively, in the case of the microemulsions.

In another study presented by Nishad et al. (2021) [69], virgin mustard oil (90% *v*/*v* of total emulsion) was enriched by dispersing grapefruit peel phenolic (GPP) extract (10% *v*/*v* of total emulsion) by preparing a w/o emulsion stabilised with high-speed homogenisation with Span-80 as emulsifier agent. Then, a nanoemulsion was prepared by means of an ultrasonication probe. The authors applied an RSM to determine the effect of emulsifier concentration (0.5–2.5%), sonication time (5–15 min) and amplitude (10–50%) on nanoemulsion physical and oxidative stability. The optimal conditions for producing a physically stable 10% GPP nanoemulsion were identified as 9.5 min of sonication at 30% amplitude with 0.52% Span-80, resulting in 29.73 ± 1.62 nm droplet sizes. These conditions improved the nanoemulsion oxidative stability (PV 35 meq/kg oil) compared to the bulk mustard oil, which had significantly higher oxidative damage (PV 65 meq/kg oil) after 30 d of storage. As for the TPC and antioxidant capacity (DPPH), the nanoemulsion system provided a protective effect on the phenolic compounds of the GPP compared to the bulk mustard oil. Thus, the nanoemulsion TPC and DPPH contents decreased 19.37% and 11.24% in storage. Meanwhile, the reduction in the mustard oil was 33.33% and 23.16%, respectively.

Al-Maqtari et al. (2021) [70] investigated the formation by ultrasound of W/O emulsion consisting of phenolic compounds from *Pulicaria jaubertii* (PJ) mixed with saline solution (22%, *w*/*w*), corn oil (70%, *w*/*w*) and employing PGPR and glycerol as emulsifiers (5% and 3%, *w*/*w*, respectively). The emulsions were prepared by high-speed and ultrasonic homogenisation, studying the influence of ultrasound power from 200 to 600 W on the physical and oxidative stability of the emulsions, among other quality factors.

Increasing levels of ultrasonic power to 600 W led to the lowest coalescence rate and particle size, thus increasing the physical stability of the emulsions. The release of TPC and oxidative stability evaluated after 28 d of storage were also improved after sonicating emulsions at 600 W, releasing approximately 91.95 mg GAE/mL emulsion, while the least stable emulsion (i.e., control without sonication) released 97.74 mg GAE/mL emulsion. These findings suggest that the 600 W sonication treatment significantly improved oil encapsulation and emulsion oxidative stability during the storage time. All emulsions showed a continuous increase in the antioxidant capacity of the oil phase, measured by DPPH, indicating that some compounds responsible for antioxidant activity had migrated from the water droplets to the oily phase. The results confirmed that both W/O 400 W and W/O 600 W emulsions presented the lowest release of antioxidant compounds, providing high encapsulation efficiency. These results agree with Robert et al. (2019) [72], who concluded that high-energy stabilisation technologies improve emulsion stability while reducing the leakage of encapsulated bioactive compounds.

The study conducted by Katsouli et al. (2018) [71] focused on optimising water-in-oil (w/o) nanoemulsions using EVOO as the continuous phase and ascorbic and gallic acids as bioactive compounds, employing RSM. The formulations tested included olive oil (92–96% *w*/*w*), Tween 20 (2–6% *w*/*w*) and water (2% *w*/*w*). Key properties evaluated were mean droplet diameter, polydispersity index (PDI), turbidity and emulsion stability index (ESI). The results demonstrated that emulsifier and bioactive compound ratios significantly influenced these properties.

The optimal formulation was achieved with 1% *w*/*w* bioactive compound (ascorbic or gallic acid) and 4% *w*/*w* Tween 20. These conditions resulted in emulsions with reduced droplet size (138–186 nm), low polydispersity index (PDI < 0.3), low turbidity (128–142 NTU) and high physical stability (ESI > 99%), ensuring resistance against destabilisation processes such as coalescence and creaming. The low turbidity values indicate a homogeneous and visually appealing appearance, an essential feature for consumer-facing applications. Gallic acid showed superior stabilising properties compared to ascorbic acid, attributed to its higher surface activity and better compatibility with the emulsifier. These optimised emulsions were effective systems for incorporating, protecting and enhancing the bioavailability of sensitive bioactive compounds in food.

Katsouli et al. (2022) [66] investigated W/O nanoemulsions where the continuous phase consisted in a mixture of EVOO and olive pomace oil (OPO), which was enriched by surface-active phenolic compounds extracted from the olive kernel. These compounds were solubilised within the water droplets and stabilised by non-ionic emulsifiers such as Span 20 and Span 80. The emulsions were formulated by homogenising the aqueous phase containing polyphenols with the oil phase and emulsifiers, followed by sonication to reduce droplet size. The study evaluated the emulsions’ physicochemical properties, including mean droplet diameter (MDD), PDI and stability during storage.

The optimised W/O nanoemulsions achieved smaller MDD values (287–590 nm) and lower PDI when phenolic compounds were incorporated and 4% Span 20 and 94% OPO were used, which enhanced droplet disruption and reduced size variability, parameters related to better physical stability. The nanoemulsions showed phase separation after 60 d of storage despite being more stable than emulsions without the olive kernel extract. However, the MDD remained stable for 30 d. Water in OPO-based nanoemulsions showed a decrease in phenolic compound retention, ranging from 46.9–54.7%. These emulsions demonstrated good physical stability with minimal droplet growth over 30 d and satisfactory antioxidant retention, highlighting their potential as delivery systems for bioactive compounds in food applications.

It is noteworthy the research conducted by Montoro-Alonso et al. (2024) [9], who evaluated the controlled release and bioaccessibility of HT and OLE encapsulated in water in EVOO emulsions, with PGPR as surfactant and emulsified by high-intensity sonication. HT exhibited a gradual and controlled release throughout the gastrointestinal phases, with over 80% bioaccessibility observed in the gastric phase and maintained stability in the intestinal phase. This sustained release was attributed to the emulsion’s ability to protect HT from premature degradation and facilitate diffusion into the aqueous phase during digestion. In contrast, OLE displayed higher bioaccessibility in the gastric phase but experienced a notable decline during the intestinal phase, potentially due to its susceptibility to enzymatic and pH changes in the latter stage of digestion. Despite this, the encapsulation process provided significant protection compared to free compounds, highlighting the W/O emulsion’s role in enhancing these phenolic compounds’ stability and overall recovery during gastrointestinal digestion.

The abovementioned research papers investigate incorporating phenolic compounds into vegetable oil emulsions and nanoemulsions to enhance their oxidative stability, phenolic content and antioxidant capacity. The studies utilised W/O emulsions, employing surfactants such as PGPR, Span 80 and Tween 20, as well as technologies like ultrasound and high-speed homogenisation. Generally, key findings of the articles include that the enriched emulsions exhibited smaller droplet sizes, lower PDI and greater oxidative resistance compared to unfortified oils. Emulsions protect bioactive compounds from degradation during storage and digestion, enhancing their bioaccessibility under intestinal conditions. However, it should be noted that the whitish appearance of some of the emulsions obtained, despite the improved physical and oxidative stabilities and enhanced bioaccessibility of the phenolic compounds, may represent a problem for the marketing of this product since it could hardly be labelled as a vegetable oil enriched in phenolic compounds, without causing rejection in the potential purchaser and consumer.

Additionally, there are other aspects to consider, such as the fact that oils enriched in phenolic compounds can be used as an ingredient for preparing food products, such as mayonnaise. For instance, Giacintucci et al. (2016) [73] used EVOO enriched with commercial phenolic olive extract at two different concentrations (0.1 and 0.2% *w*/*w*) and pure OLE (85 mg/kg) to prepare mayonnaise-like systems. The results revealed that using phenolic-enriched EVOO, especially with OLE, resulted in lower viscosity and yield stress systems, indicating they presented low resistance against deformation by shear forces (e.g., stirring). Therefore, these findings evidence that when using enriched EVOO in formulating emulsified complex food matrices, it is essential to consider both the quantity of phenolic molecules and their qualitative composition, as these compounds can influence the physical properties and stability of the emulsified structure.

More aspects to consider when incorporating nanoemulsions as bioactive compound delivery systems into food matrices is that certain compounds may alter the sensory characteristics of foods due to their intense flavours. Using emulsion and nanoemulsion systems to introduce these compounds offers an effective solution to mask undesirable off-flavours associated with their direct addition [74]. In light of this, Alemán et al. (2025) [75] assessed the effect of using W/O emulsions formulated with virgin coconut oil and quercetin-loaded chitosan nanoparticles in the sensory characteristics of enriched surimi gels. Some significant changes were seen in the sensorial attributes, such as enhanced juiciness, reduced hardness and a pronounced coconut taste and aroma. Despite these changes, most panellists highly appreciated the new product obtained with coconut oil, opening the door to launching a new product on the market with an improved nutritional profile.

## 4. Conclusions

New environmentally friendly vegetable oil extraction techniques and innovative approaches for stabilising bioactive compounds in these matrices have been reviewed to assess their applications for developing bioactive edible vegetable oil ingredients and foods. Among these, PEF technology has shown the best compromise between oil extraction yield and oil quality, especially in the production of EVOO. Cell disruption caused by PEF releases phenolic and other bioactive compounds into the extracted oil, improving its phenolic content, antioxidant properties and sensory attributes while maintaining industrial scalability. Furthermore, PEF has demonstrated versatility in other oils, such as coconut, rapeseed, sunflower and peony, consistently increasing yields and bioactive content.

In contrast, HHP exhibits high extraction rates and enhances bioactive compound content, including HT and lignans, while preserving oxidative stability and beneficial pigments like chlorophylls and carotenoids. However, its effects on oil quality are inconsistent across cultivars, emphasising the need for detailed studies to optimise process parameters based on genetic differences. UAE also offers a sustainable alternative, improving yields and enriching bioactive compounds like tocopherols and phenolic acids. Nevertheless, precise optimisation is critical to avoid oxidative degradation and maintain oil quality.

On the other hand, SWE and EAE have emerged as promising methodologies for oil extraction, offering advantages such as enhanced oil yield and the preservation of bioactive compounds. SWE has demonstrated efficacy in extracting sunflower and palm oils; however, further optimisation may be necessary for oils such as olive oil. One potential drawback of SWE is the generation of triacylglycerides during the extraction process, which can be detrimental to the quality of the final product. In contrast, EAE enhances oil quality and retains bioactive compounds, offering the added advantage of environmental friendliness by reducing harmful solvents. However, the high cost of enzymes in EAE and the temperature challenges of SWE must be addressed for broader application.

The industrial implementation of these novel food processing technologies faces significant economic, technological and regulatory challenges. One major concern is the economic feasibility since both the initial capital investment and the operational costs (i.e., energy consumption, maintenance) are normally higher than conventional methods. For instance, PEF systems require precise, high-voltage equipment while HHP or SWE involve a significant capital investment in robust pressure vessels. Moreover, adapting some of these technologies for large-scale production may be challenging. Indeed, for UAE it is difficult to achieve a uniform energy delivery in the sonication of large sample volumes. Regarding EAE technologies, their application to olive oil extraction relies on batch systems, which involve high enzyme consumption and may hinder their integration into continuous production lines. Despite these difficulties, many studies support the superior quality of the oil extracted, compared to the conventional methods. In this regard, further research is needed to evaluate the physicochemical attributes and oxidative stability of the extracted oils.

Another issue of special concern is the incorporation of active ingredients into vegetable oils. The use of delivery systems based on W/O emulsions and nanoemulsions has significantly improved oxidative stability, antioxidant capacity and bioactive compound retention in food applications. These emulsions effectively encapsulate and protect bioactive compounds such as phenolics, ensuring controlled release and enhanced bioavailability during digestion while preventing oxidative degradation. Advanced stabilisation methods, including ultrasound and high-speed homogenisation, produce emulsions with smaller droplet sizes and higher stability. However, challenges remain, such as potential consumer rejection of non-transparent emulsions and alterations in sensory properties when incorporated into food matrices. Addressing these issues and optimising formulations for commercial viability are critical for leveraging these technologies in functional food development.

Overall, this review summarises scientific data that could be transferred to the agro-industrial sector to improve production processes, meet consumer demands for higher-quality products and create new, healthier products using cutting-edge techniques for incorporating and stabilising active ingredients in oily matrices. It also highlights the use of uniform conditions in the examined research. From this point of view, it should be considered that despite the high quality of the provided results, most of the experiments were conducted under standardised conditions. In addition, for monitoring the status of bioactive compounds, studies that include individual quantification by chromatographic techniques coupled to different detectors could be considered more revealing in comparison with those that reported the total content by spectrophotometric assays. Future studies are guaranteed to evaluate the improvement of technical applications as well as the combination of new environmentally friendly vegetable oil extraction techniques and innovative approaches for stabilising hydrophilic compounds into lipophilic matrices. These studies could be addressed considering the experimental design claimed to scale up and supported by advanced analytical platforms to provide the best monitoring of nutritional profile, organoleptic properties and bioactive compounds content.

## Figures and Tables

**Table 1:** Pulsed electric field technique applied to the extraction of edible oils: technical parameters and edible oils characteristics.

Vegetable Oil	Technical Parameters		Edible Oil Characteristics				
	Set Up	Value	Oxidative Stability	Chemical Composition	Organoleptic Properties	Quality Parameters	Reference
EVOO * (Empeltre)	Electric field strength	2 kV/cm	Oxidative stability is not affected	Slight increase in sterol and phenolic contents	Not evaluated	Increase in PV * FA * and spectrophotometric indexes not modified	[15]
	Energy	39 kJ/kg					
EVOO (Tsounati, Amfissis and Manaki)	Electric field strength	18 kV/cm	Slightly improved oxidative stability	Slight increase in phenolic content	Not evaluated	Slight increase in FA PV and spectrophotometric indexes were not modified	[16]
	Energy	16, 33, 20, 50 and 70 kJ/kg					
EVOO (Koroneiki)	Electric field strength	16 kV/cm	Not evaluated	The levels of total phenolics, total flavonoids and oleuropein increased Tocopherol content significantly increased A reduction in the oleic acid content	Not evaluated	No significant effect on the saponification value, K_232_, K_270_ and ∆K	[20]
	Energy	46 kJ/kg					
EVOO (Carolea, Coratina and Ottobratica)	Electric field strength	17 kV/cm	Improved oxidative stability	Increase in phenolic content α-Tocopherol content not modified	Volatile compounds are not affected	No changes in FA, PV and spectrophotometric indices	[18]
	Energy	17 kJ/kg					
	Energy	4 kJ/kg					
EVOO (Manzanilla and Hojiblanca)	Electric field strength	2 kV/cm	Oxidative stability is not affected	Increase in phenolic content in Manzanilla cultivar α-Tocopherol content not modified	Increase in (*E*)-hex-2-enal content, but the panel test did not detect any defects	No changes in FA, PV and spectrophotometric indices	[11]
	Energy	39 kJ/kg					
EVOO (Picholine)	Electric field strength	24 kV/cm	Not evaluated	Significant increase in the total concentration of phenols, especially oleuropein derivatives α-Tocopherol content slightly increased	Volatile compounds did not show significant differences compared to control	No significant effect on, K_232_, K_270_ and ∆K, FA and PV	[19]
	Energy	4 kJ/kg					
EVOO (Nocellara del Belice)	Electric field strength	2 kV/cm	Not evaluated	Total phenolic content not affected (slight increase in oleacein and oleocanthal content)	Decrease in total alcohol content (fruity and ripe fruit attributes)	No changes in FA, PV and spectrophotometric indices	[21]
	Energy	783 kJ/kg					
	Energy	46 kJ/kg					
EVOO (Tsounati)	Electric field strength	15 kV/cm	Improvement of the oxidative stability	Higher phenolic content; 57% increase of α-tocopherol concentration compared to untreated samples	All tested samples could be characterised as EVOO and exhibited scores < 5 for fruity, bitter and pungent flavours Slight increase in bitterness	No significant effect on, K_232_, K_270_, FA and PV	[17]
	Energy	090 kJ/kg					
EVOO (Galega Vulgar)	Electric field strength	2 kV/cm	Shortened oxidative induction time compared to control, but not statistically significant	Total phenolic content non-significantly reduced Slight reduction in tocopherol concentrations	The median intensity of fruity and pungent attributes was slightly lower in the PEF sample compared with the control sample	No significant effect on K_268_, K_232_, K_270_ and ∆K, FA or PV	[22]
	Energy	85 kJ/kg					
EVOO (Coratina)	Electric field strength	21 kV/cm	Not evaluated	Improvement in total phenols of 18%	Volatile compounds did not show significant differences in sums of aldehydes, saturated or unsaturated C5 or C6 alcohols, nor C6 esters	No significant effect on K_232_, K_270_ and ∆K, FA or PV	[23]
	Energy	51 kJ/kg					
	Energy	4 kJ/kg					
EVOO (Arroniz)	Electric field strength	2 kV/cm	Not evaluated	Significant increase in the total phenolic content Significant increase in the tocopherol content Only significant differences were obtained for α-tocopherol	PEF did not affect sensory properties The median of the defects was 0	Significant increase in FA but remained below the maximum limit for EVOO under EU legislation; no significant changes in K_232_, K_270_ indices or PV	[24]
	Energy	1125 kJ/kg					
	Energy	090 kJ/kg					
Virgin coconut oil	Amplitude	40 kVcm^−1^	The iodine value was significantly higher than the control but within standard values Higher iodine values, indicate more unsaturated fatty acid content, contributing to minor oxidative storage stability	Significantly increased the total phenolic content	Not evaluated	%FFA * and PV were within standard parameters	[25]
	The distance between the two electrodes was maintained at 18 cm	Pulse width of 100 µs, a pulse off time of 50 ms and 15,000 pulses in 1232 min treatment time					
	Frequency	2560 KHz					
Rapeseed seed oil	Electric field strength	7 kV/cm	Increased oxidative stability	Increasing the intensity of the PEF in the same number of pulses, the total phenolic compounds increased compared to the control	Not evaluated	Slight increase in FA Slight reduction in PV	[26]
	Energy	3136 kJ/kg					
Sunflower oil	Electric field strength	7 kV/cm	Not evaluated	Total phenolic content increased significantly α- and γ-tocopherol content increased significantly	Not evaluated	A significant increase in FA No significant effect in PV	[27]
	Energy	61 kJ/kg					

* EVOO: extra-virgin olive oil; FA: free acidity; PV: peroxide value; FFA: free fatty acids.

**Table 2:** High hydrostatic pressure technique applied to the extraction of edible oils: technical parameters and edible oil characteristics.

Vegetable Oil	Technical Parameters		Edible Oil Characteristics				
	Set Up	Value	Oxidative Stability	Chemical Composition	Organoleptic Properties	Quality Parameters	Reference
EVOO * (Arbequina)	Energy	300/600 MPa	Oxidative stability was not affected by pressure at 600 MPa, but it decreased at 300 MPa	Decrease in phenolic content Increase in pigments (carotenoids and chlorophylls) Squalene and α-tocopherol content was not altered	Not evaluated	Increase in PV * Slight variation in spectrophotometric indexes and FA *, especially at 600 MPa	[31]
	Time	3/6 min					
EVOO (Tsounati, Amfissis and Manaki)	Pressure	200/600 MPa	Slightly improved oxidative stability	Slight increase in phenolic content	Not evaluated	FA, PV and spectrophotometric indexes not modified	[16]
	Time	1/5 min					
EVOO (Tsounati)	Pressure	600 MPa	Improvement of the oxidative stability	Higher phenolic content Increase in α-tocopherol concentration compared to untreated samples	All tested samples exhibited scores < 5 for fruity, bitter and pungent Slight increase in bitterness	No significant effect on, K_232_, K_270_, FA and PV	[17]
	Time	5 min					
EVOO (Frantoio and Moraiolo)	Pressure	600 MPa	Not evaluated	HHP pretreatment resulted in higher phenolic content in the oil	Not evaluated	No significant effect on, K_232_, K_268_, FA and PV	[33]
	Time	360 s					

* EVOO: extra-virgin olive oil; FA: free acidity; PV: peroxide value.

**Table 3:** Ultrasound-assisted extraction technique applied to the extraction of edible oils: technical parameters and edible oil characteristics.

Extraction Technique	Vegetable Oil	Technical Parameters		Edible Oil Characteristics				
		Set Up	Value	Oxidative Stability	Chemical Composition	Organoleptic Properties	Quality Parameters	Reference
UAE	EVOO * (Coratina and Peranzana)	Power output	150 W	Not evaluated	Increase in tocopherol, carotenoid and chlorophyll content Decrease in phenolic content	Reduction in bitter and pungent notes	FA *, PV * and spectrophotometric indexes not modified	[35]
		Frequency	35 kHz					
		Time	2, 4, 6, 8 and 10 min					
UAE (malaxation with oxygen control)	EVOO (Arbequina)	Power output	150 W	Improvement of oxidative stability with reduced oxygen concentration.	Increase in phenolic compounds, tocopherols, carotenoids and chlorophylls	Increase in pungency, bitterness and overall fruity attributes	Decrease in FA and PV Spectrophotometric indexes not modified	[36]
		Frequency	20 kHz					
		Energy	13.5 kJ/kg					
		Intensity	150 W/cm^2^					
		Oxygen concentration	2, 5, 10 and 21%					
UAE	EVOO (Coratina)	Power output	150 W	Not evaluated	Increase in tocopherol, carotenoid and phenolic contents	More harmonic taste: slightly less bitter and pungent	FA, PV and spectrophotometric indexes not modified	[37]
		Energy	6, 9, 12 and 15 kJ/kg					
		Time	2, 3, 4 and 5 min					
UAE	*Paeonia lactiflora* Pall seed oils	Power output	300 W	Not evaluated	Higher concentrations of some phenolics compared to hot-pressing depending on whether seeds are hulled or de-hulled	Not evaluated	FFA * and PV meet the established standards for oil quality	[38]
		Frequency	40 KHz					
		Time	60 min					
		Temperature	40 °C					
UAE	*Opuntia ficus-indica* (Sanguigna and Surfarina) Seed Oils	Frequency	40 kHz	Not evaluated	Lower tocopherol and carotenoid contents than with Soxhlet extraction	Not evaluated	FFA was not significantly affected by the extraction procedure	[39]
		Time	30 min					
		Temperature	30 °C					
UAE	Cactus seed fruit oils	Power output	150, 400 and 600 W	Iodine value increased consistently with the treatment	Phenolic content increased significantly with treatment levels, driven by a rise in canolol, while other phenolic acids remained stable or showed moderate changes α- and γ-tocopherol content significantly increased with the treatment levels	Not evaluated	A significant reduction in PV at 400 but slight increase at 600 A significant increase in FA with the treatment levels	[40]
		Time	60 min					
		Temperature	40 °C					
UAE	Favela (*Cnidoscolus quercifolius*) seed oil	Intensity	20 W/cm^2^ 28 W/cm^2^	Not evaluated	Favela oil showed up to ~60% higher tocopherol and ~24% higher β-sitosterol concentrations than Soxhlet extraction under optimal conditions	Not evaluated	Higher DPPH * antioxidant capacity compared to the oil extracted by the Soxhlet method	[41]
		Temperature	30 °C 45 °C					
		Volume-to-mass ratio	5 mL/g					
		Time	5 min					
UAE	Grape seed oil	Power output	700 W	Slightly improved oxidative stability	Higher phenolic compound content than the control sample	Not evaluated	Increase in free radicals and PV Higher antioxidant capacity by the FRAP * method than the control sample	[42]
		Frequency	20 kHz					
		Time	30 min					
		Amplitude	42 µm					

* EVOO: extra-virgin olive oil; FA: free acidity; PV: peroxide value; FFA: free fatty acids; DPPH: 2,2-diphenyl-1-picrylhydrazyl; FRAP: ferric-reducing antioxidant power.

**Table 4:** Technical parameters applied to the extraction of edible oils for Enzymatic-assisted aqueous extraction.

**Source**	Sunflower Oil	Sunflower Oil	Pecan Nut Oil	Peanut Oil	Sesame Oil	Njangsa Seed Oil	Pumpkin Seeds	Pumpkin Seeds	Tiger Nut Oil
**Extraction Technique**	EAE *	EAE	EAE	EAE	EAE	EAE	EAE	EAE	HP *-EAE
**Raw material-to-water ratio**	1:5 (g/g)	6:1 liquid/solid (mL/g)	0.4 g/mL	-	1:6 mg/mL	25 g/150 mL distilled water	-	1:2 sodium acetate buffer	1:4 mg/mL
**pH**	4.5	4.5	8	8.5	4.5 (pectinase), then 7 (alcalase)	5.0 (hemicellulose), 4.0 (protease), 4.0 (pectinase), 5.0 (amylase)	7.5	4	8 (amylase and alcalase), then 5 (celluclast)
**Temperature (°C)**	60 °C	60 °C	52 °C	60 °C	50 °C (pectinase), then 55 °C (alcalase)	55 °C (hemicellulose), 37 °C (protease), 40 °C (pectinase), 70 °C (amylase)	60 °C	50 °C	40 °C (amylase), then 50 °C (alcalase and celluclast)
**Time (h)**	5 h	2 h	4 h	3 h	8 h	24 h	16 h	24 h	6 h (EAE), 20 min (HP)
**% Enzymes**	1% (*v*/*v*)	2% (cellulase/pectinase ratio 2:1)	1.5% (Alcalase)	1.5%	10% (pectinase, alcalase)	2% (based on the weight of the flour) for all	1/100 *w*/*w* enzyme/substrate (1%)	Not indicated	0.5% (amylase, alcalase, celluclast)
**Shaking**	-	-	120 rpm	-	-	120 rpm	-	120 rpm	-
**Reference**	[43]	[46]	[47]	[48]	[49]	[50]	[51]	[52]	[53]

* EAE: enzymatic-assisted aqueous extraction; HP: hot-pressing.

**Table 5:** Technical parameters applied to the extraction of edible oils for subcritical water extraction.

Type of Oil	Pressure	Material-to-Solvent Ratio	Temperature	Time	Reference
Sunflower oil	30 bar	1:20 g/mL	130 °C	30 min	[58]
Sunflower oil	35 bar	1:20 mg/L	130–240 °C	5–120 min	[59]
Palm oil	50 bar	1:5 g/mL	130 °C	60 min	[60]
*Camellia oleifera* oil	30 bar	1:10.79 g/mL	133.59 °C	32.03 min	[61]

**Table 6:** Emulsification procedures for encapsulation of phenolic compounds in water-in-oil emulsions.

Oil Type	Emulsifier	Encapsulated Compound	Emulsification Procedure	Reference
EVOO *, olive pomace oil	Span 20, Span 80	Olive kernel phenolic extract	Coarse emulsion: olive kernel phenolic extract (0.5%) and emulsifier (Span 20 or Span 80) were mixed with water. The aqueous phase was homogenised with oil at 9000 rpm for 10 min at 40 °C. Nanoemulsion: the coarse emulsion was sonicated for 10 min at 400 W and an amplitude of 50%.	[66]
Virgin Walnut Oil, Virgin Pistachio Oil, ROO	PGPR *, Lecithin-DMG	Walnut and pistachio phenolic extracts	Emulsion: addition of 0.5–4% of phenolic extract to oil with 0.1–0.5% of PGPR using ultrasound for 30 s at 4 °C. Microemulsion: stirring oil with 1–10% of 3:2 lecithin/DMG mixture for 12 h. The aqueous phase (0.5–4.0%; milliQ water containing 100–250 mg/mL of the freeze-dried extract, and 30% propylene glycol) was added drop by drop under stirring.	[68]
Virgin Mustard Oil	Span-80	GPP * extract	Coarse emulsion: dissolving Span-80 in mustard oil at 50 °C by stirring for 30 min. The aqueous phase (10% GPP extract) was added drop by drop, and the mixture was homogenised at 10,000 rpm for 10 min. Nanoemulsion: the coarse emulsion was homogenised by ultrasound at 20 kHz and varying conditions of amplitude (20–40%) and sonication time (5–15 min).	[69]
Corn Oil	PGPR, Glycerol	PJ * phenolic extract	PGPR was mixed with corn oil, and PJ was mixed in glycerol by stirring for 5 min at 50 °C. The emulsion was homogenised at 15,000 rpm for 10 min at 50 °C and then homogenised by ultrasound at 20 kHz for 5 min at power ranging between 100 W and 600 W.	[70]
EVOO	Tween 20	Ascorbic and gallic acids	EVOO was homogenized with Tween 20 at 12,000 rpm at 40 °C. The aqueous phase (0–1% ascorbic or gallic acid in deionised water) was added at a rate of 50 μL per 30 s while homogenising.	[71]
EVOO	PGPR	Hydroxytyrosol and oleuropein	PGPR was mixed with EVOO and the olive leaf extracts were dissolved in water. The aqueous phase was dispersed into the oil phase by stirring at 300 rpm for 5 min. The emulsion was sonicated at an amplitude of 46% and a 14 mm diameter sonotrode.	[9]

* EVOO: extra-virgin olive oil; PGPR: polyglycerol polyrricinoleate; GPP: grapefruit peel phenolic; PJ: *Pulicaria jaubertii.*

## Data Availability

No new data were created or analysed in this study. Data sharing is not applicable to this article.
